# Peripheral and cardiac determinants of exercise intolerance in classic Anderson-Fabry disease: insights from cardiopulmonary exercise testing and advanced echocardiography

**DOI:** 10.1186/s13023-026-04515-y

**Published:** 2026-07-27

**Authors:** Letizia Spinelli, Alessandra Cuomo, Antonio Bianco, Eleonora Riccio, Antonella Di Donato, Antonio Pisani, Guido Iaccarino

**Affiliations:** 1 Department of Clinical Medicine and Surgery, Federico II University, Via Pansini 5, 80131 Naples, Italy; 2 Department of Public Health, Federico II University, Naples, Italy

**Keywords:** Anderson-Fabry disease, Exercise intolerance, Cardiopulmonary exercise testing, Skeletal muscle metabolism, Left atrial strain, Advanced echocardiography

## Abstract

**Background:**

Exercise intolerance is a common feature of Anderson-Fabry disease (AFD), but its underlying mechanisms remain incompletely understood.

**Methods and results:**

We studied eighty-eight patients with classical AFD phenotype and preserved ejection fraction. Patients underwent cardiopulmonary exercise testing (CPET), lactate assessment, and advanced echocardiography including myocardial work and atrial strain analysis. Exercise tolerance was reduced across the cohort, with low peak VO2 measured as percent predicted (VO2_%predicted_) and early lactate accumulation. Peak VO2_%predicted_ declined with worsening left ventricular geometry. Left ventricular diastolic parameters, myocardial work efficiency, and left atrial strain were associated with exercise capacity. In multivariable analysis, left atrial conduit strain emerged as a strong independent predictor.

**Conclusions:**

Skeletal muscle energetic metabolism alterations primarily drive exercise intolerance in AFD and early cardiac dysfunction, assessed by advanced echocardiography further exacerbated it.

**Graphical Abstract:**

**Figure:**
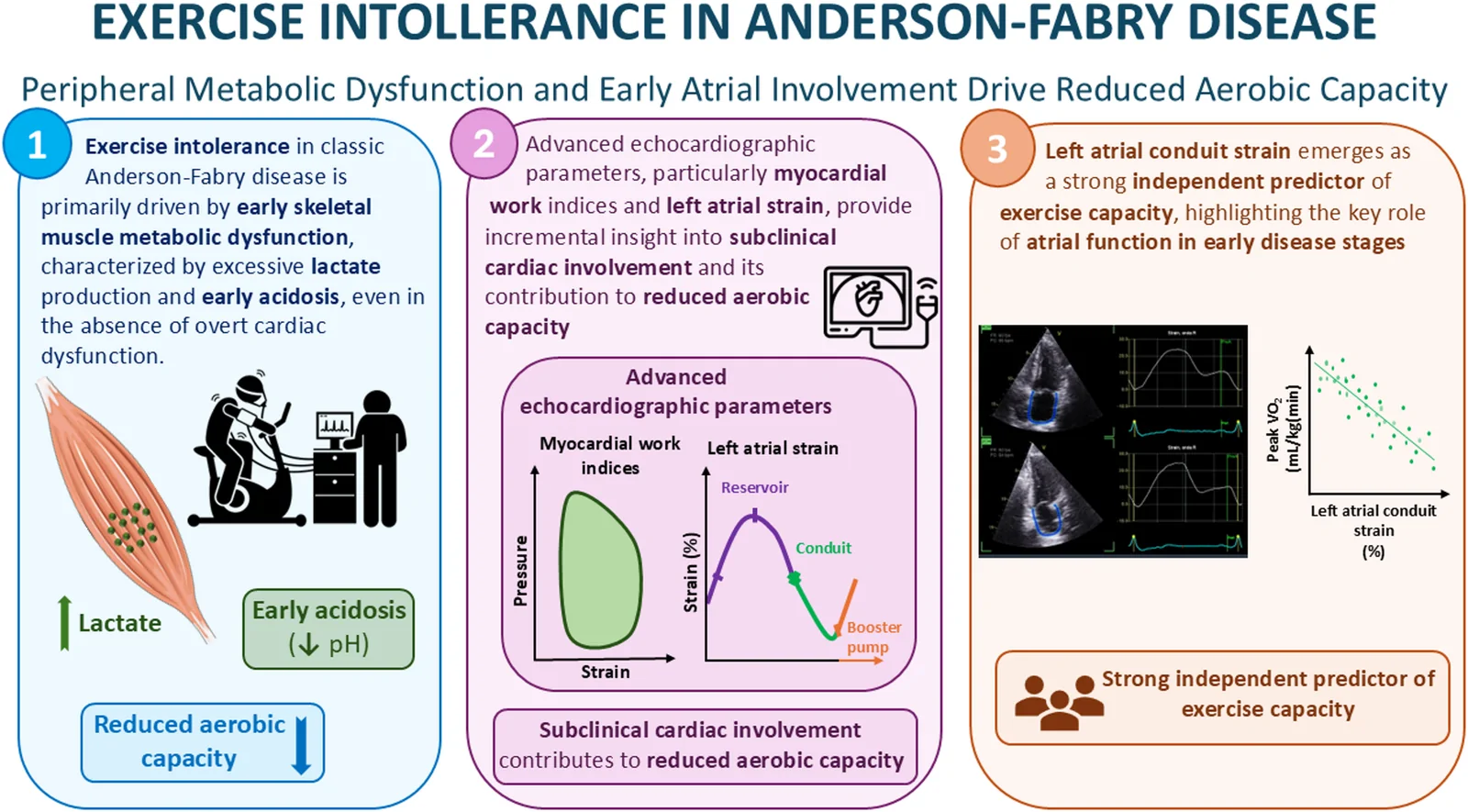

## Clinical perspective

**What is new?:**

• This study demonstrates that exercise intolerance in classic Anderson-Fabry disease is primarily driven by early skeletal muscle metabolic dysfunction, characterized by excessive lactate production and early acidosis, even in the absence of overt cardiac dysfunction.

• Advanced echocardiographic parameters, particularly myocardial work indices and left atrial strain, provide incremental insight into subclinical cardiac involvement and its contribution to reduced aerobic capacity.

• Left atrial conduit strain emerges as a strong independent predictor of exercise capacity, highlighting the key role of atrial function in early disease stages. (Graphical Abstract)

**What are the clinical implications?:**

• Evaluation of exercise intolerance in Anderson-Fabry disease should integrate cardiopulmonary exercise testing with advanced echocardiography to capture both peripheral (muscular) and central (cardiac) determinants.

• Early identification of skeletal muscle metabolic impairment may support the use of targeted interventions and improve patient management before overt cardiac dysfunction develops.

• Assessment of left atrial function and myocardial work may enhance risk stratification and guide timing of disease-specific therapies in patients with preserved ejection fraction.

## Introduction

Anderson-Fabry disease (AFD) is a rare, x-linked, genetic disorder caused by deficient α-galactosidase A (αGLA) enzyme activity, leading to progressive lysosomal accumulation of complex sphingolipids in several tissues and organs [1–3], most frequently the heart and kidney. Classical and late-onset phenotypes can be distinguished according to the onset of cardiac and renal damage [4], and the same mutation may result in different phenotypes across generations [5]. A marked clinical variability is also observed in the response to treatment, particularly regarding regression of organ damage [6, 7].

Reduced exercise tolerance and fatigue during daily activities is consistent feature of AFD, reported in both classic and late-onset phenotypes, in males and females, and independently of the αGLA mutation [8, 9]. The mechanisms underlying this limitation are multifactorial, involving cardiac and respiratory dysfunction, primary skeletal muscle abnormalities, and neurological impairment [10, 11]. Cardiopulmonary exercise testing (CPET) enables an integrated assessment of cardiovascular, respiratory, muscular, and metabolic responses to exertion [12–14], including parameters such as peak oxygen uptake (peak VO₂) and the ventilatory equivalent for carbon dioxide, expressed as the VE/VCO₂ relationship. These parameters allow stratification of prognosis and ventilation-perfusion mismatch severity [15].

Emerging evidence indicates that skeletal muscle in AFD relies predominantly on glycolytic anaerobic metabolism during maximal energetic demand. This phenotype has also been reproduced in animal models and associated with a prevalence of glycolytic muscle fibers [10, 11]. Such metabolic remodeling may directly contribute to exercise intolerance by promoting extracellular pH reduction, muscle soreness, and early fatigue.

Exercise limitation in AFD may also reflect structural and functional cardiac abnormalities such as progressive left ventricular (LV) remodeling and fibrosis, hypertrophy, and dysfunction [16–18]. While exercise intolerance is classically associated with LV systolic dysfunction, in AFD resting ejection fraction is often preserved until advanced disease stages. Global longitudinal strain (GLS), a sensitive marker of LV systolic function, may already be impaired in pre-hypertrophic stages, before ejection fraction declines [19–22]. Since GLS is influenced by loading conditions, heart rate, and LV geometry, myocardial work (MW), which integrates longitudinal deformation with instantaneous LV pressure, has been introduced to reduce load dependency [23, 24]. Growing evidence suggests that MW may allow early detection of subclinical LV systolic dysfunction in AFD [25, 26].

LV diastolic dysfunction may further contribute to reduced exercise tolerance, particularly through progressive changes in left atrial volumes and mitral annular tissue Doppler velocities [27, 28]. Recent ultrasound techniques suggest that left atrial function may be impaired in early AFD, even before measurable atrial enlargement, and may already be associated with reduced effort tolerance [29–32]. Similarly, in other forms of heart failure with preserved ejection fraction, left atrial dysfunction is independently related to impaired exercise capacity [33, 34]. Right ventricular function has also been proposed as a potential determinant of CPET performance in AFD [32, 35]. Speckle-tracking echocardiography allows a geometry-independent evaluation of right ventricular mechanics and can detect subclinical abnormalities not identified by conventional parameters [36].

Based on this background, we investigated the mechanisms of exercise intolerance in AFD before overt cardiac dysfunction becomes clinically evident. We enrolled patients without resting LV dysfunction or diagnosed heart failure. At rest, advanced echocardiography was used to assess LV diastolic function, LV systolic function including MW parameters, left atrial longitudinal strain during the different phases of the cardiac cycle, and right ventricular longitudinal systolic deformation. CPET was performed to evaluate the multiple physiological components of exercise, including lactate production, and these measures were correlated with standard and advanced resting echocardiographic parameters [37].

## Methods

### Study protocol

We retrospectively studied genetically proven patients with AFD of our AFD dedicated outpatient clinic between January 2024 and August 2025. We selected patients that had the following criteria: age ≥ 18years, ability to perform exercise stress testing, sinus rhythm, NYHA functional class ≤ 2, no valvular disease, LV ejection fraction > 50%, carrier of a classical AFD phenotype mutation of the GLA gene. According to standard practice, patients underwent a complete assessment of AFD target organs, including clinical examination, 12 leads ECG at rest and blood draw for biochemical determinations including estimated glomerular filtration rate (eGFR), and biomarkers such as Lyso- globotriaosylsphingosine (Lyso-Gb3), N-terminal brain natriuretic peptide (NT-proBNP) and high-sensitivity cardiac troponin I (hs-TnI), two-dimensional transthoracic echocardiography and symptoms limited CPET.

### Ethical approval

This study received ethical approval from the Campania 3 Ethics Committee (protocol number 138/2024), in accordance with the Declaration of Helsinki. Written informed consent was obtained from all participants or their guardians. All personal and medical information was anonymized to protect the participants` confidentiality.

### Two- dimensional transthoracic echocardiography

By using M3S probe and a commercially available system (Vivid E95, GE^®^ Vingmed Ultrasound AS, Horten, Norway) echocardiography was performed according to standard techniques [38] and including right ventricle focused apical four chamber view.

### Echocardiography analysis

Standard echocardiography analysis was performed according to current recommendations [38] as previously described [26]. A cutoff value ≤ 115 g/m2 for men and ≤ 95 g/m2 for women was used to determine normal LV mass index (LVMi). Relative wall thickness > 0.42 was scored as concentric remodeling when LVMi was within the normal range. Left atrial volume was measured just before the mitral valve opening using the biplane disk summation technique and was indexed according to body surface (normal volume < 34 mL/m2). LV diastolic function was assessed according to recommendations [27]. Speckle-tracking echocardiography analysis and strain derived MW measurements were carried out offline by using dedicated software EchoPAC^®^ version 6 (GE Vingmed Ultrasound^®^). By analyzing LV according to a 17-segment model, GLS was calculated by averaging the measurements obtained from three consecutive cardiac cycles. In this manuscript, GLS values are reported in absolute terms. MW and related indices were obtained through a combination of GLS and non-invasively estimated LV pressure curves, by assuming peak systolic LV pressure to be equal to the peak systolic blood pressure measured from brachial cuff immediately at the end of the echocardiographic study. Global work index (GWI), global constructive work (GCW), global wasted work (GWW) and global work efficiency (GWE) were calculated as previously described [26]. Right ventricle strain assessment was carried out by evaluating the average value of the longitudinal peak systolic strain only from the free wall (RV-FWS) and from all segments of the free wall and septal wall of the right ventricle (RV-GLS) as recommended by the EACVI [36]. Left atrial strain were calculated from the apical four-chamber and two-chamber views using a QRS-triggered strain curve. Atrial reservoir strain was measured at the highest average value of the strain curve. Atrial conduit strain was calculated as the difference of atrial strain value at the onset of the P wave minus atrial reservoir strain. Peak atrial contraction strain was assessed by the difference of maximum atrial contraction and atrial strain at the onset of the P wave [36].

### Cardiopulmonary exercise testing

All patients performed a multistage symptom-limited, upright bicycle exercise test. Workload started at 0 W and was increased by 10 W every 1 min. Patients were explicitly encouraged to maintain a fixed pedaling speed of 60 rounds per minute and to exercise until exhaustion (Borg > 18) [39]. Effort was quantified using peak blood pressure and heart rate, which was additionally age-normalized by calculating the percentage of the expected peak heart rate, defined as 208 minus (0.7x age in years [40]). Performance on the CPET was primarily quantified by oxygen consumption (VO2) peak using a breath-by-breath analyzer ERGOSTIK™ (Geratherm Respiratory Gmbh, Bad Kissingen, Germany) which was calibrated according to the manufacturer’s instructions prior to use. The predicted peak VO2 was calculated using the established equations [41]. To account for the effects of age and sex, normalized values were additionally assessed, i.e., for each patient, the observed value was expressed as percentage of the expected value (PeakVO2_%predicted_). Secondary CPET outcomes assessed were the VO2 at the anaerobic threshold, peak O2 pulse and ventilation/CO2 production (VE/VCO2) slope. Anaerobic threshold was determined manually using the V-slope method; when there was uncertainty, the ventilatory equivalent method was used. We also measured capillary lactate before and at the exercise peak, and the peak to rest ratio of capillary lactate in response to the maximal exercise was evaluated.

### Statistical analysis

Categorical variables are displayed as numbers (percentage), continuous variables as mean ± standard deviation when normally distributed, and as the median and interquartile range if non-normally distributed. One-way analysis of variance (ANOVA) with post-hoc Dunnett analysis, Kruskal-Wallis and U Mann-Witney tests and Chi-square test were used, as appropriate, to compare patient characteristics, imaging and CPET results across AFD groups.

The associations between CPET findings and echocardiography findings were assessed using linear regression analysis. All the tests were 2-tailed, and statistical significance was set at a p value of < 0.05. All analyses were performed using IBM SPSS V.29(IBM, Armonk, New York, USA).

## Results

Ninety AFD subjects were enrolled into the study. Two female patients were excluded because inadequate quality of echocardiographic imaging, thus, only eighty-eight patients (36% men) were included in the analysis. Patients were selected to carry a GLA mutation associated with classic phenotype. Three patients had previous history of non-sustained ventricular tachycardia, and they all carried an implantable cardioverter defibrillator. More than 50% of patients were on disease-modifying therapy, while twenty-three out forty-two therapy naïve patients started the treatment early after the study.

Based on standard echocardiography, patients were divided in three groups: **Group 1**: Normal LV geometry, (including patients with normal LVMi and relative wall thickness (≤ 0.42)); **Group 2**: Concentric remodeling, (including patients with normal LVMi and relative wall thickness > 0.42); **Group 3**: LV hypertrophy, (including patients with LVMi above the normal range by sex).

Demographic and clinical characteristics of cohort as whole and divided by groups are depicted in Table 1. Males were less abundant (18%) in Group 1, being the majority (55%) in Group 3. Similarly, mean age and body mass index increased while eGFR decreased from Group 1 to Group 3. Higher rates of NYHA class 2, hypertension, treatment with angiotensin converting enzyme inhibitors/angiotensin II receptor blockers, Ca + + channel antagonists, statins and diuretics occurred from Group 1 to Group 3. Similarly, Lyso-Gb3, HS troponin-I and NT-BNP were found to increase significantly across groups (Table 1).

**Table 1:** Characteristics of AFD cohort by LV geometry

	Whole AFD cohort (88)	Normal Geometry (39)	Concentric remodeling (16)	LV Hypertrophy (33)	*P*
Age (years)	44.02 ± 15.7	33.9 ± 12.1	45.1 ± 14.5*	57.5 ± 9.1*	< 0.001
BMI (Kg/m^2^)	26.1 ± 4.9	24.9 ± 4.6	25.1 ± 5.7	27.8 ± 4.3 *	0.028
Male gender n (%)	32 (36.3)	7 (17.9)	6 (37.5)	19 (57.6) *	0.002
Classical mutation n (%)	84 (95.4)	37 (94.8)	14 (87.5)	33 (100) *	0.170
NYHA class2 n (%)	14 (15.9)	0	0	14 (42.4) *	< 0.001
Current smoker n (%)	5 (5.6)	2 (5.1)	1 (6.2)	2 (6.1)	0.980
Hypertension n (%)	44 (50)	9 (23.1)	5 (31.2)	30 (90.9) *	< 0.001
Diabetes mellitus n (%)	2 (2.3)	0	1 (6.2)	1 (3)	0.346
Kidney Transplant n (%)	3 (3.4)	0	0	3 (9.09)	0.052
Stroke/TIA n (%)	2(2.3)	0	0	2 (6)	0.182
ICD, n (%)	3 (3.4)	0	0	3 (9.09)	0.052
Enzyme replacement therapy n (%)	38 (43.2)	12 (30.7)	4 (25)	22 (66.7) *	0.002
Chaperon therapy n (%)	7 (7.9)	4 (10.2)	3 (18.7)	0	0.078
Time under treatment (days)	2924.6 ± 1554.6	2624.2 ± 1173.9	2630.6 ± 1279.1	31196.1 ± 1823.5	0.490
Starting Therapy, n (%)	23 (26.1)	8 (20.4)	6 (37.4)	9 (27.3)	0.378
ACEi/ARB n (%)	42(47.7)	8(20.5)	6 (37.5)	28 (84.8) *	< 0.001
Beta blockers n (%)	12 (11.9)	1(7.7)	2 (12.5)	9 (17.2) *	0.001
Ca-channel Blockers n (%)	22 (25)	2 (5.1)	5(31.2) *	15 (45.4) *	0.001
Statins n (%)	15 (17)	0	0	15 (45.4) *	< 0.001
Diuretics n (%)	6 (6.8)	0	0	6 (18.1) *	0.005
Hemoglobin (g/dl)	13.2 ± 1.2	13.4 ± 0.7	13.1 ± 1.5	13.1 ± 1.4	0.463
eGFR (mL/min/1.73 m^2^)	96.82 ± 22.17	106.28 ± 16.3	100.06 ± 17.8	82.04 ± 23.5 *	< 0.001
Urine protein (mg/24 h)	205.72 ± 305.6	119.23 ± 106.9	186.14 ± 185.4	335.5 ± 464.5	0.015
Lyso-Gb3 (ng/ml)	5.94 ± 3.7	4.47 ± 2.4	5.70 ± 3.6	7.47 ± 4.2 *	0.003
Hs-troponin I (ng/ml)	10.45 ± 9.3	4.82 ± 1.7	7.66 ± 4.2	18.91 ± 10.7 *	< 0.001
NT-proBNP (ng/L)	180.412 ± 138.5	80.77 ± 32.9	162.67 ± 107.1*	314.32 ± 123.9 *	< 0.001

Anthropometric, clinical and laboratory characteristics are compared across LV geometry groups

Abbreviations: BMI – body mass index; NYHA – New York Heart †Association; TIA – transient ischemic attac;. ICD – implantable cadioverter device; : ACEi – ACE inhibitors; ARB – angiotensin II receptor blockers; eGFR – glomerular filtration rate; Lyso-Gb3 – lyso-globotriaosylsphingosine; HS – high sensitivity; NT-proBNP – N-terminal pro B-type natriuretic peptide

* *p*<0.05 vs. Normal LV Geometry; (Dunnet post hoc test)

Table 2 shows echocardiography results. LV volumes and ejection fraction were similar across the three groups, while statistically significant differences were found in LV diastolic parameters. Patients with LV hypertrophy showed lower mitral E/A ratio and higher left atrial volume, E/Ea ratio, and systolic pulmonary artery pressure than the other groups. Patients with concentric remodeling exhibited higher LVMi than normal geometry patients. As concerning LV systolic function, GLS was worse in patients with concentric remodeling than in those with normal geometry, although both groups showed similar MW parameters. However, patients with LV hypertrophy had lower GLS, GWE, GWI and GCW and higher GWW than both Groups 1 and 2. Similarly, left atrial strain during both reservoir and conduit phases was worse in LV hypertrophy group. Finally, a statistically significant difference was found in RV-GLS only when Group 3 were compared to Group 1patients.

**Table 2:** Echocardiographic results from AFD patients by LV geometry

	Whole AFD cohort (88)	Normal LV geometry (39)	Concentric remodeling (16)	LV Hypertrophy (33)	*P* value
LV EDVi (ml/m^2^)	51.2 ± 11.8	51.2 ± 10.2	46.9 ± 11.9	53.4 ± 13.3	0.232
LV ESVi (ml/m^2^)	17.1 ± 4.8	17.1 ± 4.1	15.4 ± 4.5	17.7 ± 5.6	0.319
LVEF (%)	66.5 ± 4.9	66.4 ± 4.7	66.8 ± 4.3	66.4 ± 5.7	0.960
LVMi (g/m^2^)	100.21 ± 44.7	70.60 ± 13.42	81.15 ± 14.9	144.44 ± 43.1*	< 0.001
RWT	0.46 ± 0.1	0.37 ± 0.4	0.45 ± 0.2 *	0.60 ± 0.1 *	< 0.001
LAVi (ml/m^2^)	31.1 ± 9.3	24.5 ± 4.2	31.1 ± 7.6 *	39.7 ± 7.7 *	< 0.001
Mitral E/A ratio	1.1 ± 0.3	1.2 ± 0.3	1.0 ± 0.3 *	0.9 ± 0.3 *	< 0.001
E/Ea ratio	7.59 ± 2.8	5.78 ± 1.3	6.77 ± 1.7	10.09 ± 2.8*	< 0.001
PAPs (mmHg)	28.46 ± 5.0	24.77 ± 1.2	27.25 ± 2.3*	33.42 ± 4.7*	< 0.001
TAPSE (mm)	19.3 ± 3.6	20.1 ± 3.5	20.7 ± 3.5	17.5 ± 3.2 *	0.003
GLS (%)	17.3 ± 3.8	19.5 ± 2.4	16.7 ± 2.7 *	14.7 ± 4.2 *	< 0.001
GWE (%/mmHg)	93.43 ± 4.6	95.38 ± 1.04	94.81 ± 1.9	90.45 ± 6.3*	< 0.001
GWI(%/mmHg)	1799.5 ± 450.6	1936.1 ± 355.1	2002.8 ± 348.5	1539.4 ± 481.9*	< 0.001
GCW (%/mmHg)	2053.3 ± 478.1	2194.6 ± 377.9	2252.7 ± 350.4	11789.7 ± 527.5*	< 0.001
GWW (%/mmHg)	121.91 ± 70.09	95.23 ± 34.6	108.44 ± 47.8	160.03 ± 93.3*	< 0.001
LA reservoir S (%)	29.9 ± 7.5	34.5 ± 5.5	29.2 ± 6.7 *	23.5 ± 6.6 *	< 0.001
LA conduit S (%)	-16.9 ± 7.6	-21.6 ± 5.4	-17.2 ± 7.5 *	-10.6 ± 5.3 *	< 0.001
LA contractile S (%)	-12.6 ± 5.1	-13.1 ± 5.1	-12.8 ± 3.7	-11.8 ± 5.7	0.588
RV GLS	-20.8 ± 3.9	-21.3 ± 3.5	-22.3 ± 3.8	-19.1 ± 3.9 *	< 0.001
RV FWLS	-24.1 ± 4.6	-24.4 ± 3.9	-26.5 ± 5.1	-22.2 ± 4.6 *	< 0.001

Left ventricular volumes, systolic and diastolic parameters, myocardial work indices, atrial strain, and right ventricular function are compared across LV geometry groups

Abbreviations: LV – Left Ventricle; EDVi – End-Diastolic Volume indexed; ESVi – End-Systolic Volume indexed; LVEF – Left Ventricular Ejection Fraction; LVMi – Left Ventricular Mass indexed; RWT – Relative Wall Thickness; LAVi – Left Atrial Volume indexed; PAPs – Systolic Pulmonary Artery Pressure; TAPSE – Tricuspid Annular Plane Systolic Excursion; GLS – Global Longitudinal Strain; GWE – Global Work Efficiency; GWI – Global Work Index; GCW – Global Constructive Work; GWW – Global Wasted Work; LA – Left Atrium; S – Strain; RV – Right Ventricle; FWLS – Free Wall Longitudinal Strain

*P* < 0.05 vs. Normal LV Geometry; (Dunnet post hoc test)

### Cardiopulmonary exercise test

Results of CPET are shown in Table 3. Overall, CPET were technically brought to completion, reaching maximal peak respiratory exchange ratio (RER), while VO2 peaks and workloads were significantly below the predicted values. The anaerobic threshold was anticipated at a < 37% workload, which is considered an extremely low threshold. Also, the increase in lactates raised to an average of 3-fold over the resting values. On the other hand, cardiac parameters such as peak heart rate and peak O2 pulse were well above the maximal threshold (> 70% of the predicted value). Therefore, the CPET clearly shows that acidosis that is reached exceedingly early during the effort is the most important limiting factor to exercise tolerance in patients with AFD.

**Table 3:** Cardiopulmonary exercise test results from AFD patients by LV geometry

	Whole AFD cohort (88)	Normal LV Geometry (39)	Concentric LV remodeling (16)	LV Hypertrophy (33)	ANOVA *P* value
Heart rate rest (b/min)	73.1 ± 12.3	74.9 ± 13.6	74.9 ± 7.8	69.4 ± 12.1	0.166
SBP rest (mmHg)	133.5 ± 17.6	128.1 ± 14.4	134.8 ± 15.7	139.7 ± 18.5 *	0.013
DBP rest (mmHg)	81.6 ± 9.7	79.8 ± 9.7	83.3 ± 12.5	83.1 ± 7.8	0.299
*Exercise peak*					
Exercise duration (s)	576.1 ± 187.8	620.7 ± 166.7	580.2 ± 179.7	524.1 ± 205.5	0.080
Peak workload (watts)	95.5 ± 31.4	100.4 ± 31.5	96.1 ± 30.8	89.4 ± 31.4	0.334
Peak workload_%predicted_ (%)	65.6 ± 19.1	65.7 ± 18.4	67.2 ± 17.6	64.6 ± 21.1	0.902
Heart rate peak (b/min)	134.5 ± 23.9	148.3 ± 18.2	134.5 ± 18.1	118.1 ± 22.3 *	< 0.001
Peak Heart rate_%predicted_ (%)	79.8 ± 11.3	83.4 ± 9.1	81.1 ± 7.1	74.3 ± 13.1 *	0.003
SBP peak (mmHg)	180.1 ± 30.8	172.8 ± 31.7	176.5 ± 28.4	192.1 ± 28.3	0.039
DBP peak (mmHg)	80.9 ± 15.1	79.1 ± 15.9	85.5 ± 15.1	80.2 ± 13.5	0.558
Peak/Rest lactate ratio	3.6 ± 2.8	4.1 ± 2.7	2.6 ± 1.5	3.5 ± 3.2	0.228
RER peak	1.24±0.2	1.27±0.2	1.23±0.1	1.21±0.2	0.446
VO2 peak (mL/kg/min)	15.6 ± 5.1	16.9 ± 5.2	16.5 ± 6.8	13.3 ± 3.2 *	0.008
Predicted VO2 peak (mL/kg/min)	41.1 ± 12.7	43.5 ± 15.9	39.3 ± 13.32	40.7 ± 13.7	0.901
Peak VO2_%predicted_ (%)	39.7 ± 13.7	43.5 ± 15.9	38.5 ± 13.1	38.3 ± 11.1 *	0.051
Oxygen pulse (mLO2/beat)	8.2 ± 2.3	7.6 ± 2.1	8.3 ± 2.7	8.8 ± 2.2	0.115
Peak O2pulse_%predicted_ (%)	72.4 ± 16.1	69.3 ± 13.9	73.6 ± 19.1	76.57 ± 16.8	0.150
VE peak (L/min)	42.2 ± 13.6	41.5 ± 12.6	44.4 ± 16.2	41.9 ± 13.6	0.482
VE/VO2	33.8 ± 6.3	32.1 ± 5.4	36.1 ± 6.8	34.7 ± 6.7	0.069
VE/VCO2 slope	27.2 ± 4.3	25.8 ± 4.9	29.3 ± 3.3	27.9 ± 3.6	0.105
*Anaerobic Threshold*					
Anaerobic threshold VO2 (mL/Kg/min)	10.1 ± 3.5	11.3 ± 3.7	10.1 ± 3.4	8.4 ± 3.5 *	0.002
Anaerobic threshold workload (Watts)	53.4 ± 21.2	58.7 ± 24.4	48.1 ± 12.2	49.6 ± 19.6	0.110
Anaerobic threshold workload_%predicted_ (%)	37.2 ± 15.4	38.6 ± 16.4	36.1 ± 17.6	34.9 ± 14.4	0.589

Exercise performance parameters including heart rate, blood pressure, workload, oxygen consumption, ventilation, and lactate response are reported across LV geometry groups. Abbreviations: DBP – Diastolic Blood Pressure; O2 – Oxygen; RER – Respiratory Exchange Ratio; SBP – Systolic Blood Pressure; VE – Minute Ventilation; VE/VO2 – Ventilatory Equivalent for Oxygen; VE/VCO2 – Ventilatory Equivalent for Carbon Dioxide; VO2 – Oxygen Uptake

*P < 0.05 vs. Normal LV Geometry; (Dunnet post hoc test)

By Groups, patients with AFD achieved a similar RER ≥ 1.2. Exercise duration, maximal workload, and workload at anaerobic threshold were not statistically different between the three groups. Rest and peak exercise systolic blood pressure were higher, while peak exercise heart rate was lower in Group 3 than in Group 1. Similarly, peak oxygen pulse, ventilatory volumes, VE/VCO2 slope and the increase in lactates blood levels were not different between groups. Also, PeakVO2_%predicted_ did not change from Group 1 to Group 3.

### Association between VO2 and echocardiography parameters

LV diastolic parameters, systolic pulmonary artery pressure, left atrial volume and function (both reservoir and conduit strains) were all associated to the PeakVO2_%predicted,_ by univariate regression analysis. Myocardial Work parameter, namely the Global Work Efficiency (GWE), was the only feature of LV systolic function to be found associated to PeakVO2_%predicted_. Conversely, no statistically significant association was found between right ventricle longitudinal strain and PeakVO2_%predicted_ (Table 4).

**Table 4:** Relationships between echocardiographic parameters and Peak VO2_%predicted_

	Univariate regression analysis			Multivariable regression analysis		
	B	95%CI (min/max)	*P* value	B	95%CI (min/max)	*P* value
LVMi	0.0350	0.102 / 0.032	0.303			
RWT	-9.238	-30.386 to11.889	0.387			
LAVi	− 0.335	− 0.666 to − 0.004	**0.047**	− 0.057	− 0.659 to 545	0.851
Mitral E/A ratio	12.504	3.544 to 21.463	**0.007**	6.841	-3.227to 15.898	0.154
E/Ea ratio	-1.539	-2.550 to − 0.529	**0.003**	− 0.498	-1.760 to.763	0.434
PAPs	− 0.617	-1.204 to − 0.029	**0.040**	0.860	− 0.169 to 1.888	0.100
TAPSE	0.231	0.653 to1.115	0.605			
GLS	7.350E-007	0.000 to 0.000	0.067			
GWE	0.688	0.048 to 1.327	.**035**	− 0.122	-1.065 to.822	0.798
GWI	0.001	− 0.005 to.008	0.672			
GCW	0.000	− 0.006 to 0.007	0.905			
GWW	− 0.043	− 0.085 to − 0.001	**0.045**	− 0.015	− 0.060 to.029	0.490
LA reservoir S	0.557	0.194 to.921	**0.003**	− 0.179	-849 to.491	0.596
LA conduit S	− 0.711	-1.084 to − 0.338	**< 0.001**	− 0.711	-1.084 to − 0.338	**< 0.001**
LA contractile S	0.535	− 0.053 to-1.123	0.074			
RV GLS	− 0.164	− 0.943 to.616	0.678			
RV FWLS	− 0.050	− 0.679 to − 0.579	0.876			

Univariate and multivariable linear regression analyses assessing associations between echocardiographic parameters and exercise capacity

Abbreviations: CI – Confidence Interval; LVMi – Left Ventricular Mass indexed; RWT – Relative Wall Thickness; LAVi – Left Atrial Volume indexed; PAPs – Systolic Pulmonary Artery Pressure; TAPSE – Tricuspid Annular Plane Systolic Excursion; GLS – Global Longitudinal Strain; GWE – Global Work Efficiency; GWI – Global Work Index; GCW – Global Constructive Work; GWW – Global Wasted Work; LA – Left Atrium; S – Strain; RV – Right Ventricle; FWLS – Free Wall Longitudinal Strain

By linear regression multivariable analyses including echocardiographic parameters, left atrial conduit strain was a strong predictor of PeakVO2_%predicted_ (Table 4).

## Discussion

This is the first study to integrate skeletal muscle metabolic response and myocardial work analysis to explain exercise intolerance in AFD.

The primary result is that exercise tolerance is reduced in the AFD population as whole. Indeed, all tests were maximal with RER > 1.2, and the heart rate response and peak oxygen pulse were both above 70%, suggesting that systolic function was not the major limiting factor in AFD. Our findings confirm previous observation and support the role of extracardiac factors affecting the response to exercise. Indeed, reduced exercise tolerance in AFD might be due to vascular, neurological, and working muscles abnormalities alongside cardiac and pulmonary alterations [10, 11]. We focused on skeletal muscle disturbances, which are often reported in patients with AFD. Growing evidence supports significant metabolic alterations of skeletal muscle that limit physical performance and induce fatiguability. Early involvement of mitochondrial dysfunction is a mechanism of metabolic and bioenergetic stress in AFD, according to the reduced expression of respiratory chain complexes and the decline of ATP production [42–44]. In AFD skeletal muscle, Gambardella et al. demonstrated a remodeling of the energetic metabolism towards anaerobic energetics, with accumulation of fatty acids as underutilized metabolites, and lactates as overproduced catabolites [11]. Stress-induced lactate production represents an easy-to-detect, low-cost biomarker of skeletal muscle involvement and muscular metabolic dysregulation. In our AFD cohort, stress-induced lactate levels were consistent with those previously found [11], independently from severity of cardiac dysfunction. This is indirect but consistent evidence that patients with AFD utilize glycolytic anaerobic metabolism to reach the maximal energetic expenditure. Although skeletal muscle metabolism was not directly measured, lactate kinetics provide a robust surrogate marker [11].

Exercise tolerance further deteriorated with the worsening of cardiac involvement. Indeed, on top of acidosis, the myocardial VO2 at peak exercise worsened with LV diastolic and systolic dysfunction, assessed noninvasively by MW. GWE decreased with the increase in LVMi and aging and directly correlated with PeakVO2_%predicted_. In this clinical setting, MW outperformed GLS in the assessment of LV systolic function and in predicting exercise tolerance. This prevalence of MW over GLS also occurs in another condition different from AFD, namely cardiac amyloidosis, where GWI could identify patients with poor exercise tolerance better than GLS [45]. Thus, advanced echocardiography is more sensitive to spot LV dysfunction than standard echocardiography and GLS. These findings support early functional assessment even in patients with preserved EF.

Current study highlights the association between left atrial dysfunction, and the reduced exercise tolerance as atrial conduit strain was strong predictor of peakvo2_%predicted_. Left atrial dysfunction has been recognized as a paramount pathophysiologic mechanism of reduced exercise tolerance in heart failure with preserved ejection fraction [34], with left atrial stiffness being independently associated with symptoms and quality of life [33]. In AFD hearts the left atrial reservoir, conduit, and contractile functions may all be affected, even in the early stage of the disease [29–31], when exercise tolerance is as well impaired. is intrinsically by LV functional condition influences left atrial function, given that LV systolic function contributes to reservoir phase by creating a tethering effect that facilitates left atrial passive filling. On the other hand, LV diastolic function influences transmitral filling during the passive ‘conduit’ phase. While keeping lungs free from congestion, left atrial function is of crucial importance to maintaining LV preload and cardiac output during physical activity. Decline of LV stroke volume with exercise occurs in patients with AFD with severe cardiac involvement [46].

Previous findings demonstrated a positive correlation between Peak VO2 and right ventricular volumes at cardiac magnetic resonance imaging [32, 35]. Our results failed to show any significant relationship between right ventricle function as assessed by longitudinal strain and PeakVO2_%predicted_, supporting that the role of right ventricle function in influencing exercise tolerance of patients with AFD is ancillary respect to that of left heart.

Cardiomyopathy in AFD is not a simple storage cardiomyopathy, as a 3-phase development process of the cardiac phenotype has been hypothesized: accumulation, myocyte inflammation and hypertrophy, and fibrosis/impairment. A sexual dimorphism in the myocyte hypertrophy/inflammation phase has been suggested with men experiencing a more precocious and more severe hypertrophy [47]. In female patients, X-chromosome random inactivation (lyonization) results in mosaicism, with some cells expressing the normal allele and others the mutated allele [48]. Therefore, manifestations may be heterogeneous, from an asymptomatic or mild phenotype, manifesting later in life and affecting more than one organ, to a severe phenotype resembling classic AFD [49]. The prevalence of women over the current population is higher than in previous studies [32, 35, 50] but similar to that found by Reant [51] and reflects a realistic clinical scenario [52].

It is worth considering that, our results may be influenced by sex distribution, those concerning the relationships between LV systolic function and exercise aerobic capacity. Indeed, 25 out 32 male patients (78%) presented structural LV abnormalities (concentric remodeling or concentric hypertrophy), while most women (57%) exhibited a normal LV geometry, a condition where MW is still within normal range [53]. We tried to normalize this distribution by using for our analysis a sex and age normalized parameter, the PeakVO2_%predicted_ value.

The use of medications that can affect heart rate response during exercise (e.g., chronotropic incompetence) and blood pressure, (which in turn influences Myocardial Work calculations), increases by the Group. Therefore, it is possible that the impact of cardiac damage on exercise tolerance is underestimated in Group 3, that has the most use of ACEi/ARB, Calcium antagonists and betablockers.

Our study places the altered skeletal muscle energetic metabolic as the main determinant of reduced exercise tolerance in AFD as suggested by indirect indicators, which is consistently observed across age and sex in classical patients with AFD. On top of this altered skeletal muscle alteration, we demonstrate for the first time the association of LV systolic dysfunction by GWE and the further impairment of exercise tolerance. This finding supports the usefulness of this afterload-independent evaluation of LV contractility as key component of diagnostic algorithm for heart evaluation in AFD. Additionally, we confirmed the key role of left atrial strain as predictor of exercise tolerance.

### Study Limitations

Several potential limitations should be acknowledged. Given the single center nature of the study and the rarity of disease, the number of included patients can be thought limited, compared with non-AFD studies. It should consider, however, that our cohort is larger than any other from previous studies addressing exercise performance in AFD [32, 35, 51]. Although offering useful insights into mechanisms underpinning the impairment in aerobic capacity of AFD, results cannot be generalized as patients with a more severe cardiac involvement and unable to accomplish a maximal exercise test were not included. A further limitation might arise from the fact that the potential information by myocardial fibrosis had not been addressed, since cardiac magnetic resonance was not available for every patient. In this study, no healthy control group was established, and this might be considered a limitation. To overcome this issue, we have corrected CPET results as percentage of the predicted according to age and sex, although to clearly distinguish AFD-specific skeletal muscle metabolic abnormalities and reduced exercise tolerance from the effects of general physical inactivity (deconditioning), an age- and sex-matched healthy control group would have been desirable.

## Data Availability

Data are available upon motivated and approved request by the Authors.
